# Improvement of the functionality of pancreatic Langerhans islets via reduction of bacterial contamination and apoptosis using phenolic compounds

**DOI:** 10.22038/IJBMS.2018.27718.6753

**Published:** 2018-09

**Authors:** Mahban Rahimifard, Shermineh Moini-Nodeh, Kamal Niaz, Maryam Baeeri, Hossein Jamalifar, Mohammad Abdollahi

**Affiliations:** 1 Toxicology and Diseases Group, The Institute of Pharmaceutical Sciences (TIPS), Tehran University of Medical Sciences, Tehran, Iran; 2 Department of Drug and Food, Faculty of Pharmacy, Tehran University of Medical Sciences, Tehran, Iran; 3 Department of Toxicology and Pharmacology, Faculty of Pharmacy, Tehran University of Medical Sciences, Tehran, Iran

**Keywords:** Antibacterial, Apoptosis, Ellagic acid, Islets of Langerhans, Islet transplantation Oxidative stress, Silybin

## Abstract

**Objective(s):**

During type-1 diabetes treating by pancreatic islet transplantation, increasing oxidative stress and microbial contaminations are the main reasons of transplantation failure. In this study, we evaluated anti-apoptotic, antioxidant and antimicrobial potentials of phenolic compounds called ellagic acid (EA) and silybin on rat pancreatic islets.

**Materials and Methods:**

By doing MTT assay, effective concentrations of EA and silybin were determined as 1500 and 2100 μM, respectively. Then, ELISA methods, flow cytometry and MIC were done to investigate antioxidant, anti-apoptotic and antibacterial effects of those compounds, respectively.

**Results:**

Results of FITC Annexin-V and PI staining via flow cytometry, and also caspase-3 and -9 activities performed that EA has anti-apoptotic effects on pancreatic cells. Both compounds significantly diminished reactive oxygen species, and enhanced antioxidant power and insulin secretion. Furthermore, the minimum inhibitory concentration test indicated that these two have antibacterial effects on both Gram-positive and Gram-negative bacteria which usually contaminate the pancreatic islets.

**Conclusion:**

These findings support that use of EA and silybin can improve the function of islets which are used in transplantation, along with decreasing islets bacterial contamination.

## Introduction

The rate of people, who are suffering from type-1 diabetes (insulin-dependent diabetes), increases each year. Among different procedures for the cure of this kind of hyperglycemia, pancreatic islet transplantation is an accepted method; although it has some problems during its procedure. A critical problem in islet therapy is a shortage of pancreatic islets, which is because of an imbalance between reactive oxygen species (ROS) and antioxidant defense (1, 2). 

There are a large number of polyphenols and flavonoids such as ellagitannins, ellagic acid (EA), gallotannins, proanthocyanidins, anthocyanins and silybin present in fresh and processed fruits and vegetables (3-5), which have antioxidant effects and can be used during the islet transplantation process. Previous studies indicated that polyphenols and flavonoids have anti-inflammatory, anti-apoptotic, anti-ageing, anti-diabetic, antimicrobial, gastro- and cardio-protective properties (6-14).

The polyphenol compounds, such as EA have the ability to scavenge free radicals formed during oxidative stress (15). It is believed that EA offsets the negative effects of oxidative stress by having a direct action as an antioxidant or by activating/inducing cellular antioxidant enzymatic systems (16). EA a tannin-based polyphenol is composed of dilactone (4,4΄,5,5΄,6,6΄-hexa hydroxyl diphenic acid 2,6,2΄,6΄-dilactone) and has the ability to exert antioxidant effects on the oxidative stress gene, by *cis*-acting enhancer present in the promoter region (17). The oxidative stress induces aberration of a chromosome or direct strand damage in DNA through free radicals, which were made in extracellular or intracellular pathways (18). *In vitro *studies have revealed that EA exerts growth-inhibiting and apoptosis-inducing cytotoxicity towards various cancers (19-21). Recently, EA has exhibited a dual activity; acts mostly antioxidant, although asserts pro-oxidant as well (22). This EA extraordinary property is conditional to cell type (23).

*Silybum marianum *(milk thistle) extraction contains the active constituent of silymarin, which accounts 50-75% of flavonolignan silybin, also recognized as silibinin (24). Silymarin and its derivatives have been used as medications for protection of liver (25). It has been revealed in the *in vitro* studies that silymarin has both anti-oxidant and anti-inflammatory properties, including a reserve of superoxide formation in the Kupffer cells (26, 27). Furthermore, endothelial programmed cell death induced by hydrogen peroxide can be inhibited by silybin (28). Another study showed that the programmed cell death of ECV304 cells was potentially suppressed by silybin (29). Through the process of DNA hypoploid changes, condensed nuclear chromatin, cleaved and disintegration induces apoptosis were affected by silybin. Assays such as electrophoretic mobility shift and NF-kB-dependent-luciferase showed that silybin efficiently prevents a constitutive NF-kB activation. Relying on this; the level of a nuclear p65 subunit of NF-kB was significantly reduced with silybin therapy (30). Other study showed that the uses of silymarin appear to diminish proteinuria in rat of streptozotocin-induced diabetes and in patients with type-2 diabetes (31, 32); however, the exact mechanisms are still unidentified. 

In clinical studies, islet transplantation safety and sterility of the cells are very important. But, unfortunately, microbial contamination such as viral, bacterial and fungal in islet transplantation occurs during the surgical procedure, isolation, cultivation, and transplantation of pancreatic islets. Some viruses harm body cells directly and trigger autoimmune responses in the islets, which initiate type-1 diabetes. The development of type-1 diabetes occurred when enterovirus infects β-cells (33, 34). It was shown that preservation media of pancreas has 74% Gram-positive bacteria, 21% Gram-negative bacteria, and 5% fungi (34). On the other hand, EA and silybin have already shown antibacterial properties on Gram-positive and Gram-negative bacteria (35).

With regard to the above facts, the present study aimed to evaluate the anti-apoptosis, antioxidant and antibacterial effects of EA and silybin, using half maximal effective concentration (EC_50_). It is hypothesized that these two phenolic compounds can ameliorate the oxidative stress markers and improve function of the islets by increasing insulin secretion and decreasing bacterial contaminations.

## Materials and Methods

***Chemicals***

RPMI 1640 medium, HEPES sodium salt,3-4,5-dimethylthiazol-2-yl-2,5-diphenyltetrazolium bromide (MTT), 2’,7’–dichlorodihydrofluorescindiacetate (DCFH-DA), thiobarbituric acid (TBA), 2,4,6-tripyridyl-striazine (TPTZ), N-acetyl-Asp-Glu-Val-Asp-p-nitroanilide (Ac-DEVD-qNA), N-acetyl-Leu-Glu-His-Asp-p-nitroanilide (Ac-LEHD-qNA) and Mueller Hinton Broth (MHB) were purchased from Sigma-Aldrich (Gmbh Munich, Germany). ApoFlowEx® fluorescein isothiocyanate (FITC) kit from Exbio (Vestec, Czech Republic) and rat specific insulin ELISA kit were obtained from Mercodia (Sweden).

***Animals and pancreatic islets preparation***

All experimental procedures were done under institutional review board (IRB) approval with a code number of IR.TUMS.VCR.REC.1395.464. Male adult Wistar rats (age: 2-3 months, weight: 200-250 g) were kept safe enough in the laboratory for a suitable period of time. After that, intraperitoneal injection of ketamine-xylazine in the ratio of 10:1 (100 mg/kg ketamine, 10 mg/kg xylazine) was used to anesthetize mentioned rats. After the laparotomy process and injection of Krebs buffer into the pancreatic duct, the pancreas gets baggy and easier to be separated from fatty tissues, lymph nodes, and surrounding blood capillaries. Isolated tissues were kept in Krebs buffer in order to remain healthy and prepared for the next step. The pancreas was homogenized by cutting off it into small pieces. Then after washing the chopped parts with Krebs buffer; they were centrifuged at 3000 g for 60 sec at 4 ^°^C. But islets were not completely unconnected in this level; this is why collagenase enzyme was used to make all the islets disparate. Shaking islets was done for 10 min in 37 °C bain-marie. In order to stop the digestion, bovine serum albumin (BSA) was added and then they were washed twice. In the next step, islets were picked up by stereomicroscope in which every group had similar size of islets. Finally, hale islets were cultured in RPMI 1640 for 24 hr at 37 ºC for overcoming of negative effects of these stressful procedures and restoring in a refreshed and nourished medium (36).

***Study design***

Various concentrations (10, 100, 1000 µM) of EA and silybin were made in RPMI medium culture and exposed to the islets for 24 hr at 37 ºC. Then, safety of the compounds for finding EC_50_ was investigated by MTT assay. In the following, anti-apoptosis and antioxidant properties, and also releasing insulin from the islet cells were evaluated using EC_50_ concentrations.

***Cytotoxicity assay and investigating apoptosis***

*Viability investigation by MTT assay*

Cell viability was measured by MTT assay, which was modified in our lab. Islet cells were incubated for 24 hr, while various concentrations of EA and silybin were added to them; the medium was removed and washed twice with Krebs-HEPES. Then, 20 µl of MTT (0.5 mg/ml) was added and cells were incubated for 3 hrs at 37 °C. The violet crystal was dissolved in dimethyl sulfoxide (DMSO), and after 30 min, the absorption was measured at 570 nm using an ELISA reader (37).

*Investigating apoptosis vs. necrosis by flow cytometry assay*

After 24 hr, pancreatic islets were exposed to trypsin and single pancreatic cells were isolated. After adding BSA, digestion process was blocked. Then, phosphate buffer saline (PBS) was added in order to wash the cell suspension and dual staining was done for examining apoptosis vs. necrosis by use of ApoFlowEx®FITC kit. Cells with the approximate density of 3×10^5^ cells/100 μl were incubated with 5 μl of Annexin V-FITC and 5 μl of propidium iodide (PI) at room temperature for 15 min. At the end, samples were analyzed with a flow cytometer (Mindray BriCyte E6, Shenzhen, China) (38).

***Measuring activities of caspase-3 & -9***

Caspase-3 and -9 activities were evaluated by colorimetric assay modified as mentioned, based on the identity of specific amino acid, which sequences these caspases. The tetrapeptide substrates were labeled with the chromophore r-nitroaniline (ρNA). The substrate releases ρNA upon cleavage by caspase and yellow color appears that is monitored by an ELISA-reader at 405 nm. The amount of yellow color produced upon cleavage in the sample is related to the level of caspase. Briefly, the pretreated pancreatic islets were lysed in the supplied lysis buffer and were incubated on ice for 10 min. The whole cell lysates were incubated in caspase buffer (100 mM HEPES, pH 7.4, 20% glycerol, 0.5 mM EDTA, 5 mM dithiothreitol) containing 100 mM of caspase-3 and -9 specific substrates (Ac-DEVD-ρNA and Ac-LEHD-ρNA, respectively) for 4 hr at 37 °C. Then, absorbance was measured at 405 nm. The caspase-3 and -9 activities of the treatment groups were shown as the percentage of controls which assumed 100% (39).

***Survey oxidative stress biomarkers***

*Measuring cytosolic reactive oxygen species (ROS)*

DCFH-DA was used to evaluate the level of ROS produced. Each group of islets was homogenized using extraction buffer, and then was centrifuged at 2375 g for 15 min. Then, 50 μl supernaltaint of the islet extractions was added to the mixture of 10 μl 2’,7’-dichlorodihydrofluorescin (DCFH) and 162 μl assay buffer. These solutions were incubated at 37 ˚C for 15 min. At the end, with a microplate reader, the absorbance of samples was read every 10 min up to 60 min (2).

***Determination of lipid peroxidation (LPO) level***

The amount of LPO in islets was measured using TBA. This compound reacts with malondialdehyde (MDA) that produces a complex known as TBA-reactive substances (TBARSs) which is measured spectrophotometrically. Homogenized islets were diluted using buffer saline (1:5), and then 400 ml of the aliquot was mixed with 800 ml trichloroacetic acid (TCA, 28% w/v) and the next step was to centrifuge islets at 3000 × *g* for 30 min. Then, 600 ml supernatant was added to 150 ml TBA (1% w/v). The resultant mixture was incubated in boiling water bath for 15 min and then 4 ml n-butanol was added. The mixture was again centrifuged and the absorption of the supernatant was measured at 532 nm (40).

***Total antioxidant power (TAP) assay***

TAP was determined by measuring the ability to reduce Fe^3+^ to Fe^2+^. The complex between Fe^2+ ^and TPTZ makes a blue color with absorbance at 593 nm. The following procedure leads to the preparation of the ferric reducing antioxidant power (FRAP) react: mixing acetate buffer 300 mM pH 3.6; TPTZ:10 mM in 40 mM HCl; and FeCl_3_. 6H_2_O:20 mM, in the ratio of 10:1:1 just before testing. Standard was FeSO_4_.7H_2_O: 0.1-1.5 mM in methanol. After preparing FRAP solution, 50 µl from homogenized islets was added to 1.5 ml reagent and samples were kept in 37 ºC for 30 min. At the end, concentrations were calculated by using calibration curve (36). Data were shown as mM.

***Determination of total thiol molecule (TTM)***

For determining TTM in the control and test groups, 0.6 ml Tris–EDTA buffer (Tris base 0.25 M, ethylene diamine tetra acetic acid (EDTA), 20 mM, pH 8.2) was added to 0.2 ml of supernatant and after quick vortex mixing, 40 μl 5.5′-dithiobis-2-nitrobenzoic acid (10 mM in pure methanol) was added. The final volume of this mixture was made up to 4.0 ml by an extra addition of pure methanol. After 15 min incubation at room temperature, a 10-min centrifuge was done at 3000 g and ultimately the absorbance of the supernatant was measured at 412 nm (41). Data were shown as µM.

***Functionality test***

*Insulin secretion assay*

After exposing islets to EC_50_ of EA and silybin and 24 hr incubation, cells were replaced to 1 ml Krebs medium to vials. The second step was centrifuging (3000 g for 1 min) and removing the supernatant islets incubated with 2.8 mM glucose for 30 min. After that, the vials were divided into two groups: one for adding 2.8 mM glucose (basal dose) and the other one for 16.7 mM glucose (stimulant dose). After 1 hr, the vials were centrifuged and the supernatants were collected to evaluate the insulin secretion using insulin kit according to the manufacturer’s protocol and were reported in mU/ml/mg protein/hr (38).

*Protein assay*

To determine the total protein concentration of cells, Bradford reagent was added to diluted samples and the absorbance was measured by the spectrophotometer at 595 nm after 5 min. The BSA was used as a standard.

***Minimum inhibitory concentration (MIC) for finding antibacterial effects***

Antibacterial activities of silybin and EA were tested against two Gram-positive (*Staphylococcus aureus . *ATCC 6538 and *Corynebacterium xerosis (C. xerosis) (*clinical) and three gram-negative (*Escherichia coli (E. coli) *ATCC 8739, *Pseudomonas aeruginosa *ATCC 9027 and *Salmonella typhi *ATCC 19430 bacteria. By broth micro-dilution method, MIC was measured using 96 U-shaped well plates. Serial dilution of silybin and EA were prepared by using MHB. The stock microbial suspension was also prepared in MHB from a 24 hr old culture. 

Then, an aliquot of 100 μl of twofold test strain inoculum was added to each well in order to reach the final inoculum size of 5 × 10^5^ cfu.ml^-1^ (42). Each experiment was repeated three times. MIC is the lowest concentration of the compound at which the test strain does not show noticeable growth.

***Statistical analysis***

Three independent experiments were carried out in duplicate. Data were performed as a mean ± standard error. Tukey’s multi-comparison tests were done for statistical analysis and calculation correlation. The *P*-value of <0.05 was considered significant.

## Results

***Effect of EA and silybin on cell viability***

**Figure 1:**
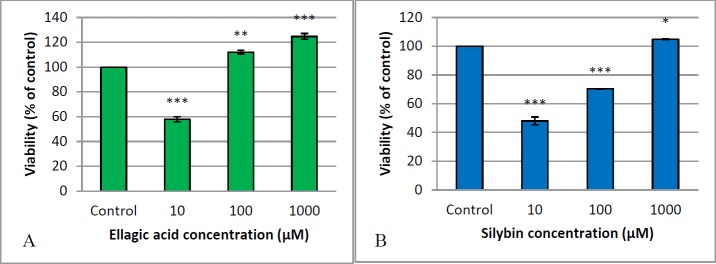
Effect of EA (A) and silybin (B) on islets cell viability. EA and silybin were administered in different concentrations of 10, 100 and 1000 µM for 24 hr at 37 ºC, and then MTT assay was done for determining EC_50_. EC_50_ of EA and silybin were calculated as 1500 μM and 2100 μM, respectively. *,**,***Significant difference from control at* p*<0.05, *P*<0.01, *P*<0.001, respectively

The outcomes of MTT assay, which report the percent of cell viability of islets, are presented in Figure 1. It shows that high concentration of both EA and silybin (1000 μM) were safe and effectively improved the viability of cells compared to the control group (*P*<0.001 and *P*<0.05). EC_50_ of silybin and EA were determined as 2100 μM and 1500 μM, respectively.

**Table 1:** Minimum inhibitory concentration (MIC) of EA and silybin against selected gram negative and gram positive bacteria

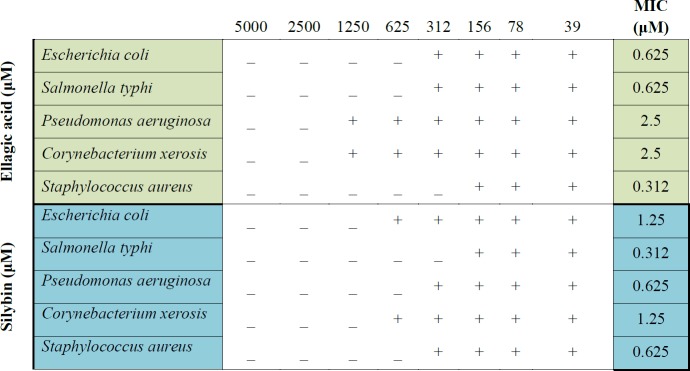

***Flow cytometry evaluation of apoptosis and necrosis***

**Figure 2:**
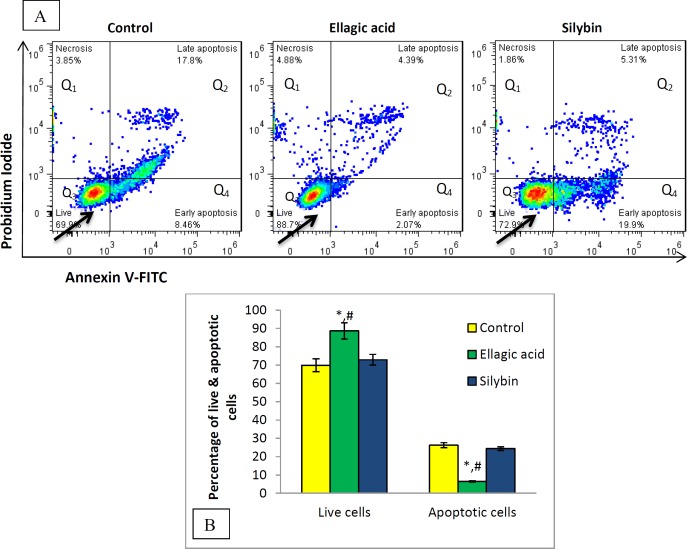
Flow cytometry assessment of effects of EA and silybin on pancreatic cells. (A) Changes in percent of live, apoptotic and necrotic populations of the islets cells are exhibited. Left down square shows live cells with FITC- and PI-, right down square indicates early apoptotic cells with FITC+ and PI-,above right square displays late apoptotic cells with FITC+ and PI+ and above left square expresses necrotic cells with FITCand PI+. (B) Percentage of live and apoptotic cells. *significant difference from control group at *P*<0.05. #significant difference from silybin group at *P*<0.05

Figure 2 illustrates the percentage of necrotic, apoptotic and viable/live pancreatic islet cells in control, EA and silybin groups. In brief, Q1 represents necrosis (FITC^-^, PI^+^), Q2 shows late-apoptotic cells (FITC^+^, PI^+^), Q3 expresses live cells (FITC^-^, PI^-^) and Q4 describes early apoptotic cells (FITC^+^, PI^-^). 

The rate of late apoptotic cells in the control group was more than EA and silybin groups. Moreover, the population of viable cells was significantly increased in EA and silybin groups up to 88.7 and 72.9%, respectively. The level of necrotic cells in silybin group was. 86%, which is less than the control and EA groups. In Figure-2B, it has been shown that EA has a higher level of live cells and less apoptotic cells compared to the control (*P*<0.05). 

***Measurement of caspase-3 and -9 activities***

**Figure 3:**
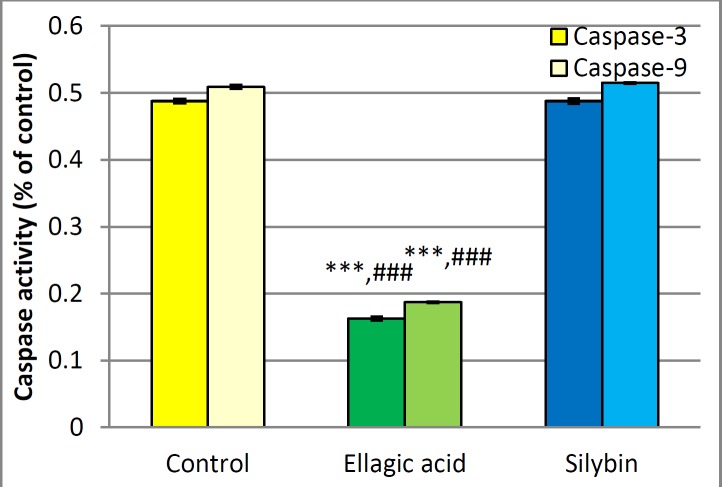
Effect of EA and silybin on caspase-3 and -9 activities on islet of Langerhans cells.***significant difference from the control group at *P*<0.001.###significant difference from silybin group at *P*<0.001

As it is shown in Figure 3, exposing islets to EA made a significant reduction in the activities of caspase-3 and -9 compared to the control group (*P*<0.001). But cells treated with silybin showed no significant changes in the activities of caspase-3 and -9.

***Oxidative stress biomarkers***

**Figure 4:**
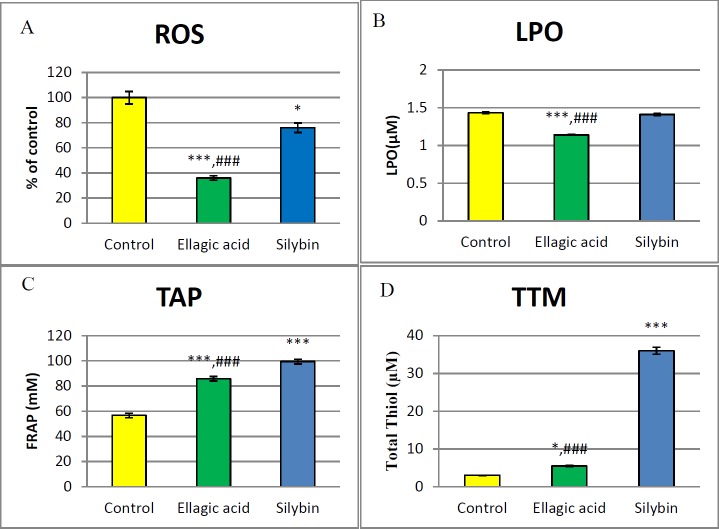
Effect of EA and silybin on oxidative stress markers. EC50 of EA and silybin were administered for 24 h. Then effects of them were evaluated on levels of (A) ROS,(B) LPO, (C) TAP and (D) TTM in islets of rats. *,***Significant difference from control at p<0.05 and *P*<0.001.###Significant difference from silybin *P*<0.001

As it is represented in Figure-4, the islet cells which were exposed to EC_50_ of EA and silybin showed a significant decrease in the level of ROS as compared to the control group (*P*<0.001 and *P*<0.05, respectively). Moreover, EA made a reduction in LPO levels (*P*<0.001).

Furthermore, silybin increased TAP and TTM production significantly (*P*<0.001). Similarly, exposure to EA made a fundamental increase in the level of TAP and TTM in the islet of Langerhans cells (*P*<0.001 and *P*<0.05, respectively).

***Insulin secretion from isolated islets***

**Figure 5:**
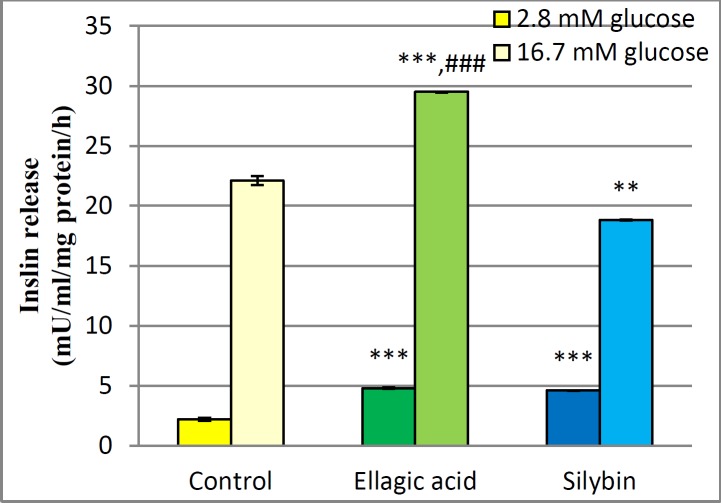
Effect of EA and silybin on insulin secretion. Islet cells were exposed to EA and silybin and incubated with 2.8 mM and 16.7 mM glucose for 1 h. **,***Significant difference from control at *P*<0.01 and *P*<0.001. ###Significant difference from silybin *P*<0.001

The effect of EA and silybin on insulin release from isolated islets in the basal and the stimulated concentration of glucose is presented in figure-5. As shown, in all groups, there is a significant increase in insulin secretion from the islets incubated with 16.7 mM glucose and those incubated with 2.8 mM. When compared with the control group, islets of Langerhans, which were treated with EA and silybin, after incubating with glucose 2.8 mm, showed a significant increment in levels of insulin.

 In addition, insulin level in the EA group incubated with 16.7 mM glucose was much more than the control and silybin groups (*P*<0.001).

***Antibacterial activities of EA and silybin***

Antibacterial properties of EA and silybin are shown in Table1. EA showed moderate antimicrobial activity against *P. aeruginosa* and *C. xerosis*, but better activity against *E. coli*, *S. typhi* (MIC: 0.625 µM) and *S. aureus* (MIC: 0.312 µM). MIC of silybin for *P. aeruginosa* and *S. aureus* was found as 0.625 µM. *S. typhi* could be inhibited by silybin at low concentrations as 0.312 µM; whereas, MIC for *E. coli* and *C. xerosis* was 1.25 µM.

## Discussion

For the last decade, phytochemicals and flavonoids have been used for medical therapy after post-genomic modernization. These flavonoids and polyphenols such as EA and silybin, are used for various advantages (43), however, they have never shown simultaneous anti-apoptotic, antioxidant and antimicrobial activities in comparative studies. Therefore, the present study, for the first time, indicates the reduction of apoptosis, oxidative stress and microbial contaminants in pancreatic islets during exposure to EC_50_ of EA and silybin. As results show, EC_50_ of EA and silybin on pancreatic islets are 1500 μM and 2100 μM, respectively.

From the current *in vitro* trail and previous studies, it has been revealed that the herbal products such as EA and silybin have hypoglycemic action in diabetic patients. The results of *in vivo* studies reported that flavonoids such as boswellic acid, EA, and rutin (rutoside) have an efficient role in reducing blood glucose level in 2 hr, except quercetin (44). The present results showed that EA and silybin have an effective role in β-cell insulin secretion. EA-treated groups, after incubating with 2.8 and 16.7 mM glucose, showed a significant higher insulin level (*P*<0.001). Moreover, in silybin group, the release of insulin from the islets incubated with 2.8 mm glucose was relatively higher than 2.8 mm glucose in the control group (*P*<0.001). Here is a comparison between flavonoids hypoglycemic activities that is as follows: rutin>quercetin>EA>boswellic acid. An *in vivo *study in streptozotocin-nicotinamide induced-diabetes showed that EA, rutin, quercetin and boswellic acid decreased the fasting blood glucose level in all diabetic and normal as compared with control group (44). The results of the EA- and silybin-treated islets showed that both compounds have a role in insulin secretion and protection of islets. In the patients with type-2 diabetes and/or streptozotocin-induced diabetes, proteinuria may be reduced after use of silymarin (31, 32); yet the exact mechanism of action is unknown. As mentioned above, in the present study, results revealed that EA and silybin have a significant role in improving pancreatic islet function.

The intracellular and extracellular production of free radicals occurs due to H_2_O_2_ damaged DNA or direct mutation in single strand; these free radicals induce oxidative stress (18). The phytochemicals such as polyphenols and flavonoids in the fruits and vegetables have the potential to decline free radicals caused by oxidative stress (15, 45). *In vitro* experiments illustrated that silybin has antioxidant and hepatoprotective effects, by reducing superoxide anion radicals and nitric oxide in the Kupffer cells (27). Additionally, programmed cell death due to hydrogen peroxide production in endothelial cells can be prohibited by silybin (28). The results of the present study showed that EA and silybin have significant antioxidant effects, and EA has anti-apoptotic effects on pancreatic islet cells. Another important finding was that EA can significantly reduce levels of ROS and LPO not only in comparison to the control group, but also when compared to the silybin group (*P*<0.001). On the other hand, interestingly silybin shows an increment in the level of antioxidant effects and total thiol molecules (*P*<0.001).

El-Shitany *et al*. (2014) presented that EA has the ability to form chelation, protect DNA damage and cellular impairment (46). *In vitro* studies show that cancer can be prohibited by inducing growth-inhibitory and apoptotic-inducing cytotoxicity through administration of EA (19-21). On the other hand, Ghasemi-Niri *et al.* (2016) stated that ulcerative colitis, which is induced by porcelain can be protected via anti-apoptotic properties of EA (47). The dual action of EA as pro-apoptosis and anti-apoptosis declares protective effects in different conditions (22). Similarly, certain dependent pathways induce apoptosis via p53-dependent way in JB6 C141 cells. Various pathways, such as protein Bcl_2_, cytochrome-c, Apaf-1, activation of caspase-3 and PARP, elaborate silymarin apoptotic effects (30). The silybin acts above the mentioned pathways, to prevent cell growth and to suppress survival of protein and mRNA expression in cancerous cells (48). Also, this study demonstrates that silybin has no antiapoptotic properties, while EA showed an anti-apoptotic effect via decreasing caspase-3 and -9 activities level (*P*<0.001) and reduction the rate of apoptotic cells in flow cytometry analysis (*P*<0.05). Increasing the rate of live cells in the islets which have been exposed to silybin was not significant.

From all microbes causing contamination, cross-examination showed that 74% of them are Gram-positive, 21% Gram-negative, and 5% fungi. The transportation time/duration of live pancreatic islets during transplantation is significantly correlated to the rate of bacterial contamination. Among microbial contamination of pancreatic islets, the potential of *Staphylococcus intermedius *growth, during preparation, transportation and transplantation is so high. Also, *Salmonella enteritidis* and *E. coli* are the most prominent organisms (49, 50). Previous study shows that EA has antibacterial effects and the MIC of that for *Fistulina hepatica* and *listeria* *manocytogenes* was 0.5 mg/ml (51). In the present trial, EA and silybin showed inhibitory effects on all microorganisms which are tested. EA showed the most antibacterial effect against *S. typhi *(0.625 μM), *E. coli* (0.625 μM) and *S. aureus *(0.312 μM), and silybin showed the best MIC against *S. typhi *(0.312 μM), *P. aeruginosa* (0.625 μM), and *S. aureus *(0.625 μM).

The widespread bacteria in the environment, which also act as a normal micro-flora of the gastrointestinal tract (GIT) in the 40-80% of people, are *Enterobacter cloacae*. Most of the other members of *Enterobacteriaceae* family also cause opportunistic infection in weakened and hospitalized patients (52). The *S. aureus* bacteria present in the extracellular space and produce pus, that’s why it is known as a pyogenic pathogen. The life-threatening diseases such as endocarditis, and confined bacterial infections like pustules, blister and abscesses come from *S. aureus*. The ability of this bacterium is related to gather in the infected tissue and initiate exotoxins, protease and enzyme. These products can spoil surrounding tissue and weaken the immune system to help the bacteria survive (53).

The microbial investigation of the pancreas showed that tissue samples taken from chronic pancreatitis or duodenopancreatectomy patients have a high amount of microbes, which deteriorates body immune system. These microorganisms, especially *Enterobacter* comes during surgery or are already present in the pancreatic duct. Usually, the fluid present in the pancreas and pancreatic duct has the potential of inhibiting the growth of *Staphylococcus* and *P. aeroginosa *(54, 55). Different studies elaborated that bacterial contamination may occur during transportation or in the isolation procedure of islet cells. 

The patient with type-2 diabetes has a low level of insulin due to post-prandial hyperglycemia. The β-cell apoptosis occurs due to glycogen breakdown in the liver, which leads to the toxic condition. Though, a high concentration of insulin has a negative impact on the β-cells physiological function and the decrease in insulin activity in body ultimately causes diabetes (56). In the present of multiple diseases, due to high pathogenic causes, chronic inflammation happens. This inflammation subsequently leads to insulin resistance (57) and pancreatic islets dysfunctionality and in the case of islet trasplantaion, results in failure (58). So, in the present study, the use of phytochemical compounds such as EA and silybin due to antibacterial and anti-inflammatory features improve the function of the pancreatic islets. 

## Conclusion

Altogether, our study declared that EA and silybin can improve the function of the pancreatic islets, inhibit bacterial contaminations and reduce apoptosis of the isolated pancreatic islets. The results display that dietary polyphenols significantly reduce LPO and ROS levels, and protect islet cells exposed to free radicals during the transplantation procedure. The anti-apoptotic effects of EA which approved by flow cytometry analysis seem to be mediated via inhibition of caspase activities. These properties of EA and silybin could be related to the phenols in their structures, which makes them antibacterial and anti-diabetic. In conclusion, EA and silybin may reduce the risk of pancreatic transplantation failure caused by oxidative stress and microbial contaminations, in type-1 diabetes patients. Of course it remains to be elucidated in clinical trials.

## Conflicts of Interest

The authors declare that they have no conflict of interest. 
